# Fibronectin Type III Domain Containing 3B as a Potential Prognostic and Therapeutic Biomarker for Glioblastoma

**DOI:** 10.3390/biomedicines11123168

**Published:** 2023-11-28

**Authors:** Hyukjun Kwon, Minji Yun, Taek-Hyun Kwon, Minji Bang, Jungsul Lee, Yeo Song Lee, Hae Young Ko, Kyuha Chong

**Affiliations:** 1 Department of Neurosurgery, Samsung Medical Center, Sungkyunkwan University School of Medicine, 81 Irwon-ro, Gangnam-gu, Seoul 06351, Republic of Korea; jess.kwon@samsung.com; 2 Photo-Theranosis and Bioinformatics for Tumor Laboratory, Research Institute for Future Medicine, Samsung Medical Center, 81 Irwon-ro, Gangnam-gu, Seoul 06351, Republic of Korea; mz.yun@sbri.co.kr (M.Y.); mj22.bang@sbri.co.kr (M.B.); 3 Department of Neurosurgery, Korea University Guro Hospital, Korea University Medicine, Korea University College of Medicine, 148 Gurodong-ro, Guro-gu, Seoul 08308, Republic of Korea; ns806@kumc.or.kr (T.-H.K.); yeosong82@gmail.com (Y.S.L.); 4 3billion Inc., 416, Teheran-ro, Gangnam-gu, Seoul 06193, Republic of Korea; jungsullee@gmail.com

**Keywords:** glioblastoma, fibronectin, biomarkers, cell signaling, in vitro techniques, survivin, STAT3 transcription factor, PTEN protein, prognosis, computational biology

## Abstract

Glioblastoma (GBM) is a representative malignant brain tumor characterized by a dismal prognosis, with survival rates of less than 2 years and high recurrence rates. Despite surgical resection and several alternative treatments, GBM remains a refractory disease due to its aggressive invasiveness and resistance to anticancer therapy. In this report, we explore the role of fibronectin type III domain containing 3B (FNDC3B) and its potential as a prognostic and therapeutic biomarker in GBM. GBM exhibited a significantly higher cancer-to-normal ratio compared to other organs, and patients with high FNDC3B expression had a poor prognosis (*p* < 0.01). In vitro studies revealed that silencing FNDC3B significantly reduced the expression of Survivin, an apoptosis inhibitor, and also reduced cell migration, invasion, extracellular matrix adhesion ability, and stem cell properties in GBM cells. Furthermore, we identified that FNDC3B regulates PTEN/PI3K/Akt signaling in GBM cells using MetaCore integrated pathway bioinformatics analysis and a proteome profiler phospho-kinase array with sequential western blot analysis. Collectively, our findings suggest FNDC3B as a potential biomarker for predicting GBM patient survival and for the development of treatment strategies for GBM.

## 1. Introduction

Glioblastoma (GBM) is currently one of the most aggressive tumors, with a median survival of 15 months even with aggressive treatment [1,2,3]. The standard therapy for GBM patients includes surgical resection and concurrent chemoradiotherapy with temozolomide (TMZ), which induces cell death by inhibiting DNA synthesis [1,4]. However, no cure or effective treatment is currently available. Due to certain molecular alterations in GBM patients, the response rate to TMZ is less than 45% [5,6]. Moreover, even patients who respond initially to temozolomide fail therapy due to acquired resistance [4,7]. Methylation of the O6–methylguanine DNA methyltransferase promoter (MGMT) is known to act as a strong drug-resistance-associated factor in GBM, and patients with isocitrate dehydrogenase (IDH) wildtype exhibit poor prognosis with standard treatment [8,9,10]. While further research is being conducted, such as studies on TMZ resistance in glioblastoma cancer stem cells, there is an urgent need to identify alternative biomarkers for GBM in light of its dismal prognosis [11,12,13].

Targeted therapies aim to inhibit specific molecular pathways dysregulated in GBM cells. For example, bevacizumab, a monoclonal antibody that targets the vascular endothelial growth factor, has been approved for recurrent GBM [14,15]. Sorafenib, an inhibitor of tyrosine protein kinases VEGFR, platelet-derived growth factor receptor (PDGFR), and Raf kinase has shown antitumoral effects in both in vitro and in vivo studies of GBM [16]. Other targeted therapies under investigation include inhibitors of the epidermal growth factor receptor (EGFR) and the mammalian target of rapamycin (mTOR) [17,18,19]. However, in contrast to specialized targeted therapies that have shown promise in the treatment of other types of cancer, these therapies have demonstrated limited efficacy in GBM. A significant challenge in developing targeted therapies for GBM is its heterogeneity, as there are many different molecular subtypes of GBM that respond differently to treatment [20,21,22]. Despite these limitations, due to the result of immunotherapy, which had been highly anticipated to be effective but failed in clinical trials, recent focuses in the development of GBM treatments have shifted to advanced personalized targeted therapies as well as vaccines, but both still require precise identification and selection of candidate biomarkers and therapeutic targets [23,24,25,26,27,28].

Fibronectin type III domain containing 3B (FNDC3B), also known as FAD104, has been previously reported as a novel gene involved in regulating the differentiation of adipocytes and osteoblasts. Among its functions, this gene functions as a positive regulator of adipocyte differentiation and as a negative regulator of osteoblast differentiation [29]. Recent research has demonstrated that FNDC3B is abnormally expressed in several types of human cancers, including hepatocellular carcinoma, acute myeloid leukemia, colorectal cancer, and cervical cancer [30,31,32,33]. For instance, overexpression of FNDC3B in hepatocellular carcinoma cell lines enhanced cell migration and invasion, suggesting that FNDC3B behaves like an oncogene and promotes tumor metastasis in hepatocellular carcinoma. This study proposes that FNDC3B could act as a potential therapeutic target and biomarker for hepatocellular carcinoma metastasis [30]. Han et al. reported that FNDC3B is upregulated in cervical cancer tissue compared with normal tissue [31]. The results also revealed that FNDC3B upregulation may be a biomarker for poor prognosis of patients with cervical cancer. Additionally, FNDC3B was found to facilitate cell proliferation and invasion via phosphoinositide 3-kinase (PI3K)/mTOR signaling and further promote colorectal cancer progression [33]. In myeloma cells, FNDC3B proteins interact with tumor suppressors FAM46C and p62 proteins and integrate protein and secretory homeostasis [34]. In GBM, a recent study reported that FNDC3B expression is upregulated, and increased expression is associated with poor prognosis [35]. Wang et al. reported that certain microRNA could suppress FNDC3B in glioblastoma, which might be one of the potential mechanisms for inhibiting the malignant phenotype of GBM cells [36]. Although FNDC3B is attracting attention as a significant biomarker in other cancer types, its biological function and usefulness as a biomarker in glioblastoma are still unclear. This study aims to investigate the role of FNDC3B in GBM malignancy and assess its potential utility as an alternative biomarker.

## 2. Materials and Methods

### 2.1. Gene Expression and Prognosis Analysis

Expression profiles of the *FNDC3B* gene and the analyzed data were collected by searching for ‘Fibronectin Type III Domain Containing 3B’ (Entrez Gene ID: 64778) in the array- and sequence-based patient transcriptomic database, ‘Oncopression’ [37]. The data in Oncopression were analyzed with the single-sample normalization method [38]. All samples used for the analysis were taken from the same Affymetrix Human Genome U133 Plus 2.0 (GPL570 or A-AFFY-44) platform. Expression values ranged between 0.0 and 1.0, with 1.0 signifying full transcriptional activation. In order to calculate the ‘cancer-to-normal’ ratio, expression values were divided by the mean normal expression values obtained from a total of 19,435 cancer samples and 4547 normal samples. The expression level of *FNDC3B* in various brain pathologies was analyzed using a total of 2510 samples. For prognosis analysis, brain tumor datasets containing expression profiles and patient prognostic information were collected. For the analysis of *FNDC3B*-dependent overall survival of the patients with high or low expression of *FNDC3B*, the level of expression was determined using the mean *FNDC3B* expression value. All gene expression values were quantile normalized by datasets. Z-values calculated from a log-rank test for each dataset were averaged by the Lipták method using the square root of the patient number of each dataset as the weight [39].

### 2.2. Cell Cultures

U87MG, T98G, PC-3, and MCF7 were acquired from the Korea Cell Line Bank (Seoul, Republic of Korea), and U87-IDH mutant and U251 were purchased from the American Type Culture Collection (ATCC, Manassas, VA, USA). The cells were cultivated using Dulbecco’s Modified Eagle’s Medium (DMEM; Catalog No. LM001-10; Welgene, Daegu, Republic of Korea) or RPMI1640 (Catalog No. LM011-02; Welgene), enhanced with 10% Fetal Bovine Serum (FBS; Catalog No. 16000044; Gibco, Grand Island, NY, USA) and 1% penicillin-streptomycin (PS; Catalog No. 15070063; Gibco), in a 5% CO_2_ incubator at 37 °C.

### 2.3. Small Interfering RNA Transfection

Specific small interfering RNAs (siRNAs) for FNDC3B and the scrambled non-specific control siRNA were purchased from Bioneer (Daejeon, Republic of Korea). For transfection experiments with Lipofectamine 2000 (Catalog No. 11668019; Invitrogen, Waltham, MA, USA), the cells were seeded into six-well plates at 2 × 10^5^ cells per well to achieve 60–70% confluence after overnight growth. Lipofectamine-siRNA complexes were prepared according to manufacturer instructions. Transfection efficiency and cancer progression-related validation were analyzed 48 h later.

### 2.4. Cell Viability Assay

To determine proliferation activity, cell viability was assessed for each group using a colorimetric assay, which evaluated metabolic changes in a water-soluble tetrazolium salt (WST-1; Catalog No. EZ-3000; DoGenBio, Seoul, Republic of Korea). The viability of U87MG cells was examined under various conditions. The assays were conducted by directly adding WST-1 to the culture wells and incubating them at 37 °C for 30 min. Subsequently, the absorbance was measured at 450 nm with a 96-well microplate reader (Catalog No. 1681150; Bio-Rad, Hercules, CA, USA). The cell viability was calculated by comparing the absorbance in the treated groups to the control group. The data were normalized by the measured absorbance of the control group.

### 2.5. Quantitative Reverse Transcription Polymerase Chain Reaction

Total RNA was extracted using the RNeasy Mini Kit (Catalog No. 74104; QIAGEN, Hilden, Germany). This total RNA (0.5–1 μg) was subjected to reverse transcription using the PrimeScript RT Reagent Kit (Catalog No. RR037A; TaKaRa, Tokyo, Japan), as per the manufacturer’s protocol. Quantitative reverse transcription polymerase chain reaction (qRT-PCR) was performed in a final volume of 10 μL, which contained cDNA and each primer set. This was performed using the QuantStudio 6 Real-Time PCR system (Applied Biosystems, Waltham, MA, USA) with a thermocycler profile of 95 °C for 10 min, followed by 40 cycles of 95 °C for 5 s, 58 °C for 25 s, and 72 °C for 30 s. The primer sequences used for qRT-PCR are listed in Table S1. The mRNA expression was normalized to that of the glyceraldehyde 3-phosphate dehydrogenase (GAPDH).

### 2.6. Cell Migration Assays

A wound-healing assay was conducted using μ-Dish 35-mm-high culture inserts (Catalog No. 81176; Ibidi, Martinsried, Germany), following the manufacturer’s instructions. Briefly, a day before the experiment, U87MG cells were seeded into each culture insert and left to incubate overnight. On the following day, the culture inserts were delicately removed using tweezers, thereby creating a gap devoid of cells between the adhered ones. Migration into this space was observed after 12 h. The images were analyzed using ImageJ (Version 1.53t; National Institutes of Health, Bethesda, MD, USA) imaging analysis software to calculate a percent wound closure.

Cell migration ability was also assessed using transwell chambers (24-well plate, 8 μm pore size; Catalog No. 3464; Corning, Corning, NY, USA). U87MG cells were suspended in a serum-free medium and adjusted to 40,000 cells/mL. DMEM, supplemented with 10% FBS and 1% PS, was introduced into the bottom of the 24-well plate. Subsequently, 100 μL of the cell suspension was added to the upper chamber of the migration well. After incubating for 24 h at 37 °C, the non-migrated cells on the top side of the membrane were carefully removed with a cotton-tipped applicator, and the cells that had migrated to the underside of the membrane were fixed with 4% paraformaldehyde for 10 min. These cells were then washed with phosphate-buffered saline (Catalog No. LB004-02; Welgene) and stained with a 0.25% solution of crystal violet (Catalog No. 5265; Sigma-Aldrich, St. Louis, MO, USA) for 10 min. The number of migrating cells was assessed by counting the cells that penetrated through the membranes in three random fields per chamber, using a light microscope (Catalog No. CKX41; Olympus Life Science, Tokyo, Japan)

### 2.7. Cell Adhesion Assay

Transfected U87MG cells were detached using 0.25% trypsin-EDTA (Catalog No. 25200056; Gibco) and resuspended in serum-free DMEM media at a concentration of 0.8 x 10^6^ cells/mL; 150 μL of this cell suspension was added to wells that had been pre-coated with various extracellular matrix proteins (Catalog No. CBA-070; Cell Biolabs, San Diego, CA, USA). After an incubation period of 90 min, the wells were washed five times with Dulbecco’s phosphate-buffered saline (Catalog No. LB001-02; Welgene), to which 2 mM each of calcium chloride and magnesium chloride had been added. Then, 200 μL of cell stain solution was applied. The stained cells were incubated on an orbital shaker for 10 min. The cells were then washed with deionized water. Subsequently, 200 μL of extraction solution was added, and the cells were again incubated on an orbital shaker. After another 10 min, 150 μL of each extracted sample was transferred to a 96-well plate, and the absorbance was measured at 560 nm.

### 2.8. Three-Dimensional Spheroid Invasion Assays

For the invasion assay, a three-dimensional spheroid invasion assay was performed with spheroids formed by seeding 2000 cells into a 96-well round bottom plate (Catalog No. 7007; Corning) for 2 days. Afterwards, these spheroids were implanted into 2.5% Matrigel (Catalog No. 356234; Corning). Invasion was measured after 1–5 days using a light microscope. Five spheroids per group were used to analyze the invasion. Cells were imaged every 24 h to determine cell invasion and migration.

### 2.9. Proteome Profiler Phospho-Kinase Array

Total protein was extracted with RIPA Lysis buffer (Catalog No. MB-030-0050; Rockland Immunochemicals, Limerick, PA, USA)) in the control and experimental groups. After protein quantification, 200 μg of each protein lysate were incubated overnight at 4 °C and subsequent experiments were performed according to the protocol of Proteome Profiler Human Phospho-Kinase Array Kit (Catalog No. ARY003C; R&D Systems, Minneapolis, MN, USA). Chemiluminescence expression pixel densities on membranes were visualized by the Amersham Imager 600 (GE Healthcare, Chicago, IL, USA) chemiluminescent imager and analyzed using ImageJ.

### 2.10. Western Blot Analysis

To yield whole cell lysates, cells were lysed using RIPA buffer with protease and phosphatase inhibitors; 10–30 μg of protein extract were resolved via SDS-PAGE and transferred to PVDF membranes. After blocking, the membranes were incubated with primary antibodies against FNDC3B (1:1000 *v*/*v*; Catalog No. HPA007859; Sigma-Aldrich), Survivin (1:1000 *v*/*v*; Catalog No. ab76424; Abcam, Cambridge, UK), Vimentin (1:1000 *v*/*v*; Catalog No. sc-80975; Santa-Cruz, Dallas, TX, USA), Twist (1:1000 *v*/*v*; Catalog No. ab50887; Abcam), Nestin (1:500 *v*/*v*; Catalog No. 611658; BD Biosciences, Frankiln Lakes, NJ, USA), BMI-1 (1:1000 *v*/*v*; Catalog No. ab126783; Abcam), signal transducer and activator of transcription 3 (STAT3) (1:1000 *v*/*v*; Catalog No. 4904T; Cell Signaling Technology, Danvers, MA, USA), phospho-STAT3 (1:1000 *v*/*v*; Catalog No. 9145T; Cell Signaling Technology), PTEN (1:1000 *v*/*v*; Catalog No. 9552S; Cell Signaling Technology), PI3K (1:1000 *v*/*v*; Catalog No. 4257T; Cell Signaling Technology), phospho-PI3K (1:1000 *v*/*v*; Catalog No. 4228T; Cell Signaling Technology), phospho-Akt (1:1000 *v*/*v*; Catalog No. 13038S; Cell Signaling Technology), Akt (1:1000 *v*/*v*; Catalog No. ab32505; Abcam), c-Myc (1:1000 *v*/*v*; Catalog No. 9165T; Cell Signaling Technology), and β-actin (1:3000 *v*/*v*; Catalog No. sc-47778; Santa-Cruz), followed by incubation with secondary antibodies conjugated to horseradish peroxidase (Catalog No. 1706515 and 1706516; Bio-Rad). β-actin was used as a loading control.

### 2.11. Network Analysis Using the MetaCore Database

Potential signaling and comparative networks of FNDC3B and associated genes were constructed using the MetaCore database (Clarivate Analytics, London, UK), an integrated pathway bioinformatics analysis platform [40,41]. The network was scrutinized with an auto-expand building algorithm comprising 50 nodes and employing canonical pathways to discern functional relationships between genes. Finally, the networks were utilized to identify potential signals related to FNDC3B.

### 2.12. Statistical Analysis

Data were analyzed using GraphPad Prism version 10.0 software (GraphPad Software, Boston, MA, USA) and are presented as means ± standard deviations. Comparisons among the groups were made using an unpaired two-tailed *t*-test with Welch’s correction (Welch’s *t*-test) for normally distributed data and with the Mann–Whitney non-parametric test for abnormally distributed data. The Shapiro–Wilk tests were employed to determine the normality of data. A *p* value of < 0.05 was regarded as statistically significant and was presented as *p* < 0.05 (*), *p* < 0.01 (**), *p* < 0.001 (***), and *p* < 0.0001 (****). For survival curve comparisons, the log-rank (Mantel–Cox) and hazard ratio (Mantel–Haenszel) tests were used.

## 3. Results

### 3.1. FNDC3B Expression and Related Prognosis Analysis Using the Oncopression Database

#### 3.1.1. *FNDC3B* Expression in Various Organ Cancers

**Figure 1:**
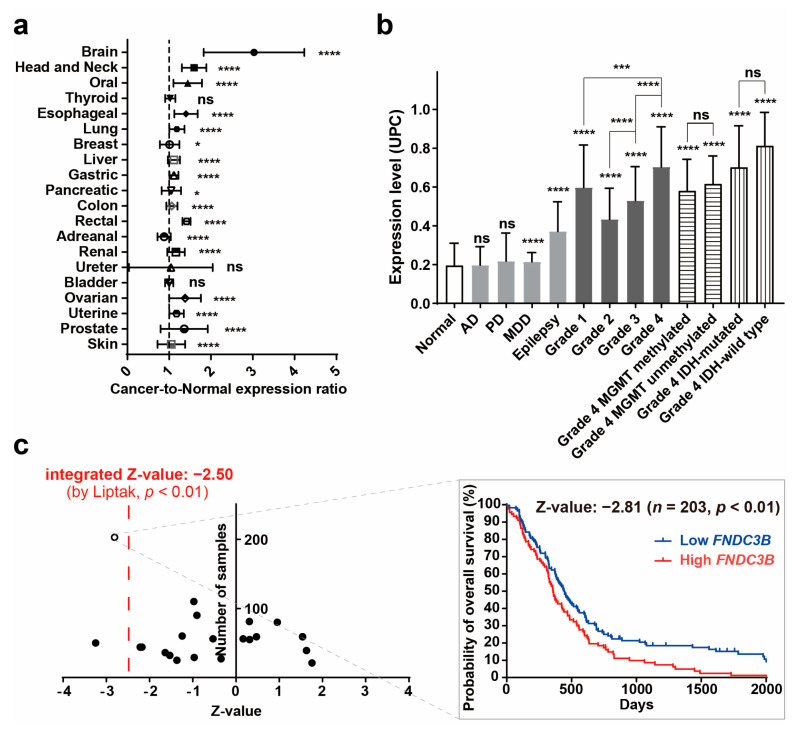
*FNDC3B* expression in human brain pathologies. (**a**) The cancer-to-normal ratio of *FNDC3B* expression in the 20 organs based on the Oncopression database analysis. The data are presented as the means ± 95% confidence interval and *p* value evaluated by unpaired two-tailed Mann–Whitney *t*-test. (**b**) *FNDC3B* expression in various brain pathologies. Expression levels of brain pathologies, including brain tumors with grades and molecular specifications, were compared with normal brains. The data are presented as Universal exPression Codes (UPC) values and analyzed by an unpaired two-tailed Mann–Whitney *t*-test. (**c**) Relationship between *FNDC3B* expression and prognosis of glioblastoma patients. A total of 1374 patient samples from 23 Grade 4 brain tumor datasets where glioblastoma is located were analyzed. The negative Z-value indicates a worse prognosis. The integrated Z-value of −2.50 was obtained using Lipták’s method. The black circles indicate the Z-value of each dataset, and a Kaplan–Meier survival curve was generated from the representative TCGA database (empty circle in the graph), which had the largest sample number and a Z-value of −2.81. * *p* < 0.05, *** *p* < 0.001, **** *p* < 0.0001, ns: not statistically significant. AD: Alzheimer’s dementia; IDH: isocitrate dehydrogenase; MDD: major depressive disorder; MGMT: O6-methylguanine-deoxyribonucleic acid methyltransferase promoter; PD: Parkinson’s disease; UPC: Universal exPression Codes value.

In an investigation of *FNDC3B* expression in normal and cancer tissues from patients, the Oncopression database was utilized to analyze the cancer-to-normal ratio of *FNDC3B* in a total of 23,982 samples across various organs. Out of 20 organs, a statistically significant difference in *FNDC3B* expression in cancer tissues was observed in 17 organs as compared to normal tissues, and no such difference was observed in the thyroid, ureter, and bladder cancers (Figure 1a, Table 1). Among the 17 organs with a statistically significant difference, 16 exhibited an increase in *FNDC3B* expression in cancer tissues, the exception being adrenal cancer, which displayed a decrease. Notably, the cancer-to-normal ratio of *FNDC3B* expression within brain cancer exceeded 3.0 (*p* < 0.0001), significantly higher than the ratios in all other organs, which remained below 1.6.

#### 3.1.2. *FNDC3B* Expression in Human Brain Pathologies and Brain Tumors

To specifically verify the expression of *FNDC3B* in brain pathologies, we compared expression levels across 2510 samples, which included Alzheimer’s dementia, major depressive disorder (MDD), epilepsy, Parkinson’s disease, and brain tumors of varying grades and molecular specifications (Figure 1b, Table 2). In non-tumor pathologies, *FNDC3B* expression showed no significant difference in Alzheimer’s dementia tissues and Parkinson’s disease tissues compared to normal brain tissue. However, there was a statistically significant increase in MDD (1.1-fold, *p* < 0.0001) and a notable increase in epilepsy tissues (1.9-fold, *p* < 0.0001). Within brain tumor tissues, we observed that *FNDC3B* expression levels progressively increased from Grade 2 to Grade 4 (Grade 2, 2.2-fold, *p* < 0.0001; Grade 3, 2.7-fold, *p* < 0.0001; Grade 4, 3.6-fold, *p* < 0.0001). Interestingly, Grade 1 exhibited a higher fold-change (3.1-fold, *p* < 0.0001) than Grades 2 and 3. This observation may be due to the fact that Grade 1 brain tumors, such as pilocytic astrocytoma, are more frequent in pediatric patients than adults, and the expression of *FNDC3B* is also partially related to angiogenesis. As per our observation of molecular biological status, *FNDC3B* expression was universally increased in Grade 4 where glioblastoma is included, regardless of whether the groups were MGMT methylated (3.0-fold, *p* < 0.0001) or unmethylated (3.2-fold, *p* < 0.0001), IDH-mutated (3.6-fold, *p* < 0.0001) or IDH-wild type (4.2-fold, *p* < 0.0001). These results indicate that *FNDC3B* is highly expressed in brain tumors, particularly those classified as Grade 4, irrespective of molecular status.

#### 3.1.3. *FNDC3B* as a Prognostic Factor for Glioblastoma

Additionally, to investigate the impact of *FNDC3B* expression on the prognosis of GBM patients, we performed prognostic analysis using quantile normalization values from the datasets. Overall survival analysis was performed on 1374 patient samples from 23 datasets available to collect patient prognostic information and high and low expression groups based on *FNDC3B* mean expression values. The integrated Z-value of *FNDC3B* expression in GBM was −2.50 (*p* < 0.01), indicating that high *FNDC3B* expression in GBM patients had worse overall survival prognosis (Figure 1c, Table S2). These results reinforced the possibility that *FNDC3B* plays a role as an oncogene and prognosis biomarker in GBM, as suggested, and prompted us to further investigations.

### 3.2. FNDC3B Expression and Relations with Survivin Expression in GBM Cells

**Figure 2:**
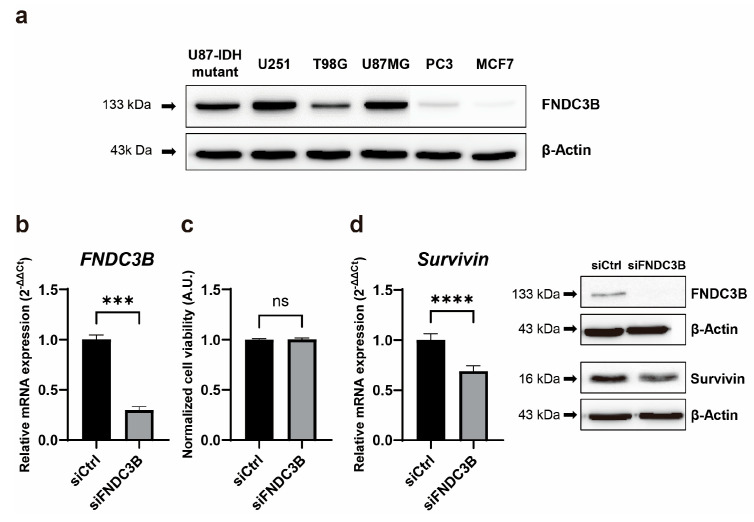
High FNDC3B expression in GBM cells and Survivin downregulation with FNDC3B silencing. (**a**) FNDC3B expression in various cancer cell lines. (**b**) Expression of *FNDC3B* in U87MG cells transfected with scrambled non-specific control siRNA (siCtrl) or FNDC3B specific siRNA (siFNDC3B) (*n* = 3). (**c**) Cell viability in transfected U87MG cells by WST-1 assay (*n* = 4). (**d**) The expression of *Survivin*, an apoptosis inhibitor, on the same parallel cells was measured by qRT-PCR (*n* = 6), and the protein level of Survivin was determined via western blot on control and FNDC3B silenced cells. β-Actin was used as a loading control. Data are presented as mean value ± standard deviations. *** *p* < 0.001; **** *p* < 0.0001; ns: not statistically significant. FNDC3B: fibronectin type III domain containing 3B.

To validate the results of the Oncopression database analysis of FNDC3B, we examined its expression in various cell lines of GBM, prostate cancer, and breast cancer. According to the Oncopression database analysis, brain, prostate, and breast cancers demonstrated cancer-to-normal ratios of 3.0 (*p* < 0.0001), 1.4 (*p* < 0.0001), and 1.0 (*p* < 0.05), respectively. In line with these results, western blot analysis indicated that while FNDC3B overexpression was observed in all GBM cell lines (U87-IDH mutant, U87MG, U251, T98G), its expression was relatively low in cell lines from prostate cancer (PC3) and breast cancer (MCF7) (Figure 2a). To investigate the function of FNDC3B in GBM cells, we constructed three types of FNDC3B-siRNA (siFNDC3B) and a scrambled non-specific control siRNA (siCtrl), tested FNDC3B gene silencing on U87MG cells (Figure S1), and used the most effective siFNDC3B (siFNDC3B No. 1, Table S3) in the subsequent experiments to investigate FNDC3B function. Using the selected siFNDC3B, we also identified a successful decrease in mRNA expression of FNDC3B with qRT-PCR analysis (siCtrl: 1.00 ± 0.08; siFNDC3B: 0.30 ± 0.06; *p* < 0.001, Welch’s *t*-test) (Figure 2b). To assess the role of FNDC3B on GBM cell proliferation, we performed a cell viability assay using WST-1 on FNDC3B-silenced U87MG cells. The U87MG cells treated with siFNDC3B exhibited no specific decrease in WST-1 assay compared with control siRNA-transfected cells (siCtrl: 1.00 ± 0.01; siFNDC3B: 1.00 ± 0.03; not statistically significant, Welch’s *t*-test) (Figure 2c). However, siFNDC3B significantly decreased *Survivin*, a well-known apoptosis inhibitor [42], compared to the group treated with control siRNA (siCtrl: 1.00 ± 0.06; siFNDC3B: 0.69 ± 0.05; *p* < 0.0001, Welch’s *t*-test) (Figure 2d). This result suggests that, although the suppression of FNDC3B alone may not directly impact cell proliferation, it may potentially enhance the anticancer effect by amplifying the apoptosis of cancer cells in treatments using anticancer drugs, such as TMZ, when FNDC3B is suppressed.

### 3.3. Inhibition of Cell Migration through FNDC3B Silencing in GBM Cells

**Figure 3:**
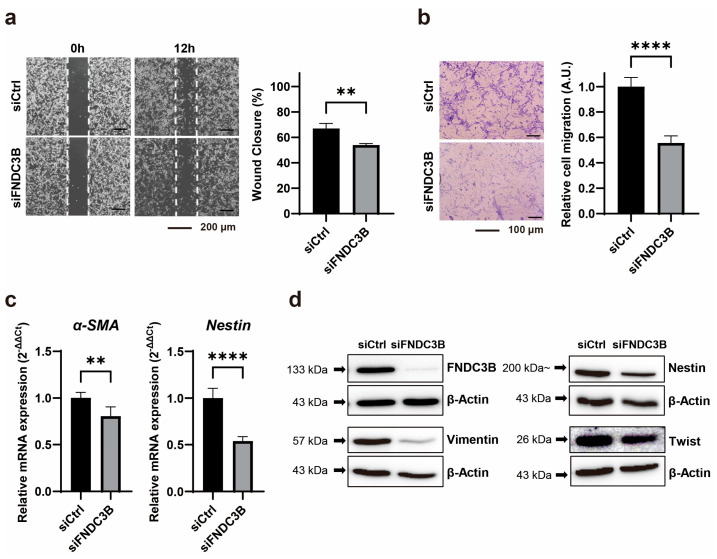
FNDC3B silencing reduces cell migration ability and epithelial–mesenchymal transition markers in GBM cells. (**a**) A wound healing assay was conducted on scrambled non-specific control siRNA (siCtrl) or FNDC3B-specific siRNA (siFNDC3B) transfected U87MG cells, 12 h after creating a gap as a wound (40× magnification; *n* = 4). (**b**) Transwell assays were performed on U87MG cells following transfection with siCtrl or siFNDC3B (100× magnification; *n* = 6). (**c**) The expression of epithelial–mesenchymal transition markers, *α-smooth muscle actin*, and *Nestin* in control cells and FNDC3B downregulated cells was measured by qRT-PCR (*n* = 6). (**d**) The protein levels of additional epithelial–mesenchymal transition markers, Nestin, Vimentin, and Twist, in control cells and FNDC3B downregulated cells were evaluated via western blot analysis. Data are presented as the mean value ± standard deviations. ** *p* < 0.01; **** *p* < 0.0001. α-SMA: α-smooth muscle actin; FNDC3B: fibronectin type III domain containing 3B.

To investigate the impact of FNDC3B silencing on GBM cell migration, we conducted cell migration assays, including a wound-healing assay and a transwell assay, on U87MG cells transfected with siRNA. We observed that FNDC3B silencing impeded the wound healing properties of U87MG cells compared to control cells (siCtrl: 67.08 ± 3.91; siFNDC3B: 54.05 ± 2.23; *p* < 0.01, Welch’s *t*-test) (Figure 3a). To verify FNDC3B-mediated alterations in GBM cell migration, we performed an additional assay using a transwell. We also noted that cell migration ability was inhibited in siFNDC3B-transfected cells compared with the control (siCtrl: 1.00 ± 0.07; siFNDC3B: 0.56 ± 0.06; *p* < 0.0001, Welch’s *t*-test) (Figure 3b). We subsequently performed qRT-PCR for *α-smooth muscle actin* (*α-SMA*) and *Nestin*, both of which are significantly regulated during the epithelial–mesenchymal transition (EMT) process (Figure 3c). The expression of *α-SMA* (siCtrl: 1.00 ± 0.06; siFNDC3B: 0.80 ± 0.10; *p* < 0.01, Mann–Whitney *t*-test) and *Nestin* (siCtrl: 1.00 ± 0.10; siFNDC3B: 0.54 ± 0.05, *p* < 0.0001; Welch’s *t*-test) was downregulated at the mRNA level, which indirectly suggests that FNDC3B silencing inhibits the EMT process. We also observed that the expression of representative molecular markers of EMT, such as Vimentin and Twist, as well as Nestin, was reduced after FNDC3B silencing in U87MG cells, as shown by western blot analysis (Figure 3d). These data provide evidence that FNDC3B modulates U87MG GBM cell migration and EMT regulation.

### 3.4. Inhibition of Cell Invasion and Stemness through FNDC3B Silencing in GBM Cells

**Figure 4:**
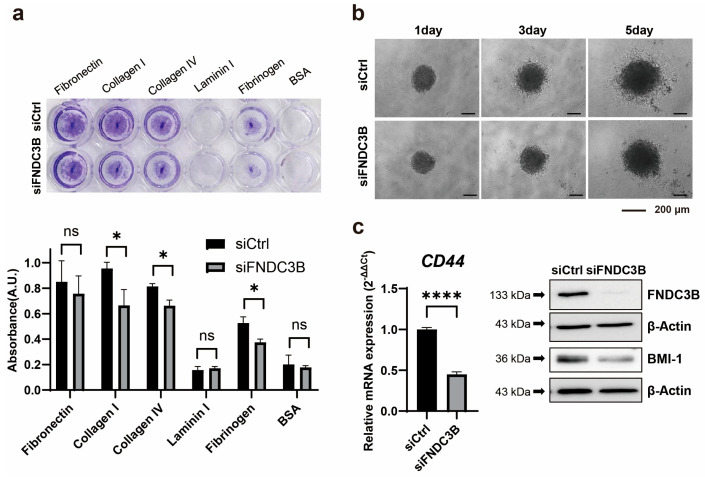
FNDC3B silencing diminishes GBM cell invasiveness and stemness characteristics. (**a**) Extracellular matrix adhesion assay on U87MG cells transfected with scrambled non-specific control siRNA (siCtrl) or FNDC3B-specific siRNA (siFNDC3B) (*n* = 3). Cells adhering to various extracellular matrix components were stained with crystal violet and quantified at 560nm after extraction. (**b**) Representative phase contrast images of a U87MG three-dimensional spheroid embedded in Matrigel and observed over 5 days (40× magnification). (**c**) The expression level of the stemness marker, CD44, on U87MG cells with siCtrl- and siFNDC3B-transfection was measured by qRT-PCR (*n* = 6), and the protein level of another stemness marker, BMI-1, was verified by western blot analysis. Data are presented as mean value ± standard deviations. * *p* < 0.05; **** *p* < 0.0001; ns: not statistically significant.

Further to identifying that FNDC3B silencing is associated with cell migration abilities and the EMT process, we investigated the effect it might have on the ability to adhere to the extracellular matrix of GBM cells. We observed that adhesion ability was reduced in siFNDC3B-transfected cells compared with siCtrl-transfected cells, significantly with collagen type I (siCtrl: 0.96 ± 0.05; siFNDC3B: 0.66 ± 0.12; *p* < 0.05, Welch’s *t*-test) and IV (siCtrl: 0.81 ± 0.02; siFNDC3B: 0.66 ± 0.04; *p* < 0.05, Welch’s *t*-test), and fibrinogen (siCtrl: 0.53 ± 0.05; siFNDC3B: 0.37 ± 0.03; *p* < 0.05, Welch’s *t*-test) (Figure 4a). Based on the correlation results between FNDC3B and cell migration, EMT process, and ECM adhesion ability, which were confirmed through FNDC3B silencing, we conducted a three-dimensional (3D) spheroid invasion assay. This was conducted to verify the relationship between cell invasiveness and FNDC3B in an additional step. For generating spheroids, an equal number of transiently transfected cells were seeded in a 96-well round bottom plate and embedded into Matrigel to perform the invasion assay. Since Matrigel is primarily composed of laminin and collagen type IV, it was used as a matrix. After 24 h, the tumor spheroids extended multicellular strands of cells from the margin into the Matrigel, displaying a typical invasion pattern called “starburst” [43]. Cells were radially spread over a period of 5 days. Elongated protrusions of FNDC3B-silenced spheroids were significantly inhibited compared to control spheroids (Figure 4b). As previous studies have reported that the acquisition of invasiveness in cancer cells is accompanied by stemness features [44,45], we analyzed the expression of *CD44* and BMI-1, universal markers for stem cells, using qRT-PCR and western blot analysis (Figure 4c). Both markers were significantly downregulated in FNDC3B-depleted U87MG cells compared with control cells, thereby indicating the relation between FNDC3B and U87MG cell stemness. This finding was consistent with the results of the current study, indicating that FNDC3B silencing also has a regulatory effect on GBM cell invasiveness and stemness properties.

### 3.5. Investigation of FNDC3B-Related Signaling Pathways in GBM

**Figure 5:**
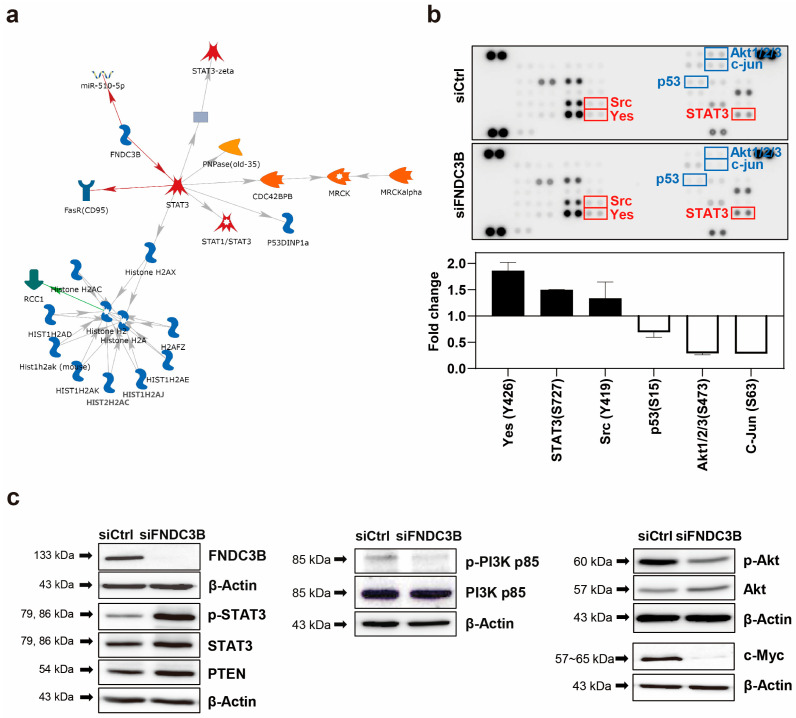
FNDC3B-related signaling pathway in GBM cells. (**a**) Network analysis results of FNDC3B and related genes using the MetaCore database, an integrated pathway bioinformatics analysis platform. A canonical pathway-based auto-expand building algorithm was used to analyze the network, which contained 50 nodes (green arrows: positive/activation effects; red arrows: negative/inhibition effects; gray arrows: unspecified). (**b**) The screening results of changes in phospho-protein activation in control and FNDC3B siRNA transfected U87MG cells were obtained using the proteome profiler phospho-kinase array. Chemiluminescence expression pixel densities were quantified with ImageJ software to analyze fold-changes in the proteins that showed alterations between the control group and the FNDC3B siRNA transfected group overall. (**c**) Phosphorylation and total expression of proteins, such as signal transducer and activator of transcription 3 (STAT3), total phosphatase and tensin homolog (PTEN), phosphoinositide 3-kinase (PI3K), Akt, and c-Myc, were examined in both control and FNDC3B siRNA transfected U87MG cells.

To investigate the FNDC3B-related mechanism of the migration and invasion of GBM cells, we conducted an analysis using the MetaCore database, which analyses protein networks, metabolic pathways, and maps for the list of genes and proteins obtained from experimental high-throughput data [46]. As a result of the MetaCore analysis, we determined that STAT3 was predominantly related to FNDC3B in network analysis, and the results suggested that FNDC3B has inhibitory effects on STAT3 (Figure 5a). Furthermore, to screen potential downstream signaling pathways induced by the FNDC3B-knockdown on GBM, we conducted a proteome profiler phospho-kinase array and analyzed protein phosphorylation profiles using the Proteome Profiler Human Phospho-Kinase Array Kit (Figure 5b). Among them, the phosphorylation of the three proteins, namely Yes (Y426), STAT3 (S727), and Src (Y419), significantly increased, while those of p53 (S15), Akt1/2/3 (S473), and c-Jun (S63) notably decreased upon FNDC3B silencing compared with the control (Figure 5b).

Given the high alteration of expression in the phosphorylation of STAT3 and Akt1/2/3 between the control and siFNDC3B treated groups, we hypothesized that FNDC3B might have a relationship with the phosphatase and tensin homolog (PTEN)-mediated PI3K/Akt signaling pathway. To verify this hypothesis, we investigated the phosphorylation and total expression of the proteins with western blot analysis (Figure 5c). The results showed that STAT3 phosphorylation and the total protein expression of STAT3 and PTEN significantly increased with FNDC3B silencing. The role of STAT3 in GBM pathogenesis is still under debate, as it is unclear whether it is pro-oncogenic or tumor-suppressive [47,48], and recent reports suggest a feedback activation mechanism in STAT3, rather than inhibition, in response to the inhibition of the PI3K/Akt/mTOR signaling pathway [49]. Therefore, we conducted additional investigations on the PI3K/Akt signaling pathway to more clearly understand how the cell signaling pathway dampens cell migration, invasion, and stemness after FNDC3B silencing. Overall, even though the total protein expression had no noticeable change and phosphorylation slightly decreased in PI3K with FNDC3B silencing, the phosphorylation of Akt significantly decreased even though the total protein expression of Akt increased. These findings were compatible with U87MG cell migration, invasion, and stemness results. Furthermore, we verified the validity of the PTEN and PI3K/Akt results by observing the reduction of c-Myc, known to inhibit PTEN [50], through FNDC3B silencing. Through advanced MetaCore database network analysis of the proteins corresponding to the results, it was possible to reconfirm that all six proteins evaluated by proteome profiler phospho-kinase array and the proteins evaluated by western blot analysis had a direct or at least indirect relationship with FNDC3B (Figure S2).

## 4. Discussion

GBM, the most common primary brain tumor, is a disease to which the upfront applications of molecular biology and genomics are critical in clinical fields, and the importance of discovering more efficient novel alternative biomarkers as prognosis predictors and therapeutic targets continue to grow [1,51,52]. Despite the advances achieved in understanding GBM molecular characteristics, effective treatments are yet to be established. Since 2005, the current standard treatment for newly diagnosed GBM has been maximal safe resection followed by radiotherapy, in combination with concomitant and maintenance of TMZ [53]. However, no other therapeutic intervention has been proven to prolong overall survival.

Recently, FNDC3B has been reported to be highly expressed in various cancers, including GBM, and to have an oncogenic effect based on TCGA and GEO databases [54]. FNDC3B was revealed to have a significant correlation between its expression and clinical prognosis, protein phosphorylation, and immune cell infiltration. In this study, we investigated the expression of FNDC3B in cancers of 20 different organs and the overall survival related to *FNDC3B* expression levels in GBM using Oncopression database analysis to validate the potential of FNDC3B as an effective prognostic and therapeutic biomarker for GBM. We found that *FNDC3B* was exceptionally highly expressed in GBM compared to other organ cancers and that the expression levels of *FNDC3B* increased along with the grade of brain tumors. In addition, survival data indicated that GBM patients with high *FNDC3B* expression had a worse prognosis than those with low expression. Based on the results, FNDC3B could be interpreted as an efficient candidate for prognosis and a therapeutic biomarker.

Compared to previous studies about FNDC3B in GBM, which only validated the results through data analysis or experiments about simple phenotypical changes related to FNDC3B, this study reports experimentally evaluated mechanisms related to FNDC3B for the first time. To investigate the role and related cell signals of FNDC3B in GBM, we performed an in vitro study, including qRT-PCR, western blot analysis, proteome profiler phospho-kinase array, and MetaCore integrated pathway bioinformatics analysis. To the best of our knowledge, the first specific mention of the relationship between FNDC3B and GBM was a report by Xu and colleagues. They reported that microRNA-129-5p effectively inhibited GBM cell proliferation, migration, and invasion by downregulating FNDC3B [55]. However, their primary focus on FNDC3B was on interpreting the effects of microRNA-129-5p on GBM cells without a specific investigation or analysis of the changes in cell signaling associated with FNDC3B. Regarding issues related to this study, their research mainly presented the results of cell viability, wound healing, and transwell assays conducted after downregulating FNDC3B using microRNA-129-5p and an FNDC3B interference RNA lentiviral vector. The findings from Xu’s study are consistent with those observed in this study, where a decrease in GBM cell migration, invasion, and stemness was noted following FNDC3B silencing using siRNA. However, unlike Xu’s research, this study did not observe any significant differences in cell proliferation following FNDC3B silencing. Nevertheless, this study identified a significant decrease in Survivin, a representative apoptosis inhibitor, in response to FNDC3B silencing. This finding is regarded as important evidence suggesting the possibility of considering FNDC3B as a target for combined therapies aimed at inhibiting GBM cell progression.

To confirm in vitro data and to define the further mechanism of FNDC3B in GBM malignancies, we analyzed an FNDC3B-related network of various molecules using the MetaCore database analysis platform. From the perspective of analyzing the network associated with FNDC3B, this analysis showed that FNDC3B has negative/inhibition effects on STAT3, STAT3 has positive/activation effects on PTEN, and PTEN has negative/inhibition effects on Akt. FNDC3B has been reported to suppress invasion and metastasis by inhibiting the phosphorylation of STAT3 in melanoma cells [56]. On the contrary to melanoma cells, this study showed that increased STAT3 phosphorylation by FNDC3B silencing could play a role in suppressing cell migration, invasion, and stemness in GBM cells. Considering the dual role of STAT3 as either a pro-oncogene or tumor-suppressor, subject to the genetic background of the tumor [47,57], FNDC3B downregulation might induce upregulation of STAT3 phosphorylation and PTEN expression solely in U87MG cells treated with siFNDC3B constructed in this study. However, as previous studies reported that STAT3 positively regulates PTEN through miR21 [58] and that PTEN inhibits cell growth and metastasis by regulating the PI3K/Akt pathway in GBM [59], this study addresses the evidence that downregulation of FNDC3B exerted the suppressive effects on the activation of PI3K/Akt signaling in GBM and that suggests a possibility of FNDC3B as a therapeutic biomarker. Nonetheless, conclusive evidence is required to determine whether such observed STAT3 alterations were not only provoked by feedback mechanisms but also facilitated by FNDC3B. It is also important to investigate if the PTEN/PI3K/Akt pathway activation in GBM was substantially mediated by STAT3.

This study has limitations. First, the study was conducted using U87MG cells as a primary test subject because the U87MG cell line is a representative cell line in GBM research. However, further assays and experiments need to be performed on various GBM cell lines to validate and gain a clearer understanding of the role of FNDC3B in GBM. The second limitation is that we only confirmed the regulatory effect of FNDC3B silencing on GBM cell stemness properties using the three-dimensional spheroid invasion assay and a few representative markers, and we did not further investigate the alteration of stemness characteristics using other methods, such as the oncosphere assay. To validate the effects of FNDC3B on GBM stemness, additional experiments and studies should be conducted.

## 5. Conclusions

Overall, these studies suggest that FNDC3B may play a role in GBM malignancies, and the findings propose FNDC3B as a potential prognosis biomarker, suggesting that targeting FNDC3B could be a promising strategy for GBM treatment. However, there is currently no clinically available drug targeting FNDC3B specifically. Thus, there is a need for preclinical studies aimed at developing small molecule inhibitors, monoclonal antibodies, and other targeted therapies capable of blocking FNDC3B or its downstream signaling pathways. We are planning and conducting further studies on FNDC3B. We believe the exploration of FNDC3B-mediated or related signaling associated with GBM progression can yield significant outcomes potentially contributing to GBM treatment.

## Figures and Tables

**Table 1:** ‘Cancer-to-Normal’ ratio of *FNDC3B* expression in various organ cancers.

Organ	Number of Cancer Samples	Number of Normal Samples	‘Cancer-to-Normal’ Ratio	*p* Value
Brain	2517	723	3.0287 ± 1.2028	<0.0001
Head and Neck	360	119	1.5969 ± 0.2944	<0.0001
Oral	309	167	1.4436 ± 0.3410	<0.0001
Thyroid	338	197	1.0261 ± 0.1225	ns
Esophageal	120	21	1.4027 ± 0.2779	<0.0001
Lung	2502	650	1.1854 ± 0.1820	<0.0001
Breast	5516	471	1.0113 ± 0.2339	<0.05
Gastric	934	110	1.1142 ± 0.1079	<0.0001
Liver	524	322	1.1138 ± 0.1453	<0.0001
Pancreatic	240	98	1.0509 ± 0.2307	<0.05
Colon	2449	500	1.0653 ± 0.1315	<0.0001
Rectal	205	21	1.4152 ± 0.0964	<0.0001
Adrenal	355	50	0.8813 ± 0.1592	<0.0001
Renal	504	195	1.1643 ± 0.2109	<0.0001
Ureter	58	45	1.0410 ± 1.0008	ns
Bladder	260	11	1.0001 ± 0.0995	ns
Ovarian	1146	92	1.3800 ± 0.3783	<0.0001
Uterine	387	192	1.1760 ± 0.1723	<0.0001
Prostate	257	75	1.3595 ± 0.5624	<0.0001
Skin	454	488	1.0501 ± 0.3296	<0.0001

The ‘Cancer-to-Normal’ ratio values are represented as means ± standard deviations of arbitrary units. *FNDC3B*: Fibronectin type III domain containing 3B, ns: not statistically significant.

**Table 2:** *FNDC3B* expression in normal brain and various brain pathologies.

Pathology	Number of Samples	Mean	Standard Deviation	Contrast to Normal (Fold)	Contrast to Normal, *p* Value
Normal	723	0.1948	0.1155	-	-
AD	227	0.1966	0.0954	1.0096	ns
PD	51	0.2177	0.1447	1.1179	ns
MDD	134	0.2145	0.0472	1.1011	<0.0001
Epilepsy	43	0.3716	0.1525	1.9079	<0.0001
Grade 1	74	0.5973	0.2193	3.0665	<0.0001
Grade 2	134	0.4330	0.1606	2.2234	<0.0001
Grade 3	132	0.5297	0.1756	2.7197	<0.0001
Grade 4	865	0.7034	0.2075	3.6113	<0.0001
Grade 4 MGMT methylated	44	0.5797	0.1633	2.9764	<0.0001
Grade 4 MGMT unmethylated	34	0.6155	0.1447	3.1600	<0.0001
Grade 4 IDH- mutated	12	0.7016	0.2143	3.6020	<0.0001
Grade 4 IDH- wild type	37	0.8123	0.1725	4.1705	<0.0001

MGMT: O6-methylguanine-DNA methyltransferase, IDH: isocitrate, ns: not statistically significant.

## Data Availability

The data presented in this study are available upon request from the corresponding author.

## Supplementary Materials

The following supporting information can be downloaded at: https://www.mdpi.com/article/10.3390/biomedicines11123168/s1, Figure S1: Western blot analysis of constructed small interfering RNAs (siRNAs) for FNDC3B downregulation; Figure S2. Network Analysis Results of FNDC3B Related to STAT3 and Akt; Table S1: Primer sequences used for real-time quantitative reverse transcription polymerase chain reaction; Table S2: Z-values related to FNDC3B expression in the database of glioblastoma patients; Table S3: Sequences of small interfering RNAs created to investigate the functions of FNDC3B.
