# Supplementary material for: Fibronectin Type III Domain Containing 3B as a Potential Prognostic and Therapeutic Biomarker for Glioblastoma
Source: Biomedicines. 2023 Nov 28;11(12):3168. doi: 10.3390/biomedicines11123168 (PMC10741045; doi:10.3390/biomedicines11123168)
Supplement: Supplementary file 1 [file biomedicines-11-03168-s001.zip › biomedicines-2688161-supplementary.pdf]

Supplementary Figures and Tables

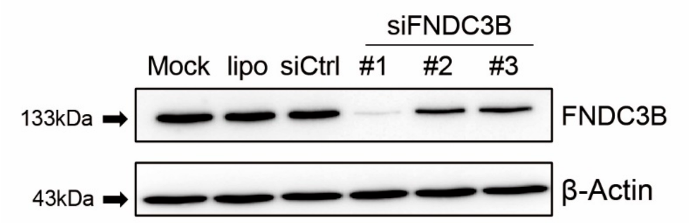

Figure S1. Western blot analysis of constructed small interfering RNAs (siRNAs) for FNDC3B downregulation. A total of three siRNAs were transfected into U87MG cells, and western blot was performed 48 hours after transfection. siFNDC3B#1 was finally selected for FNDC3B silencing as it most efficiently down-regulated FNDC3B. FNDC3B: fibronectin type III domain containing 3B; lipo: a control where only Lipofectamine 2000 was used; siCtrl: scrambled non-specific control siRNA; siFNDC3B: FNDC3B-siRNA.

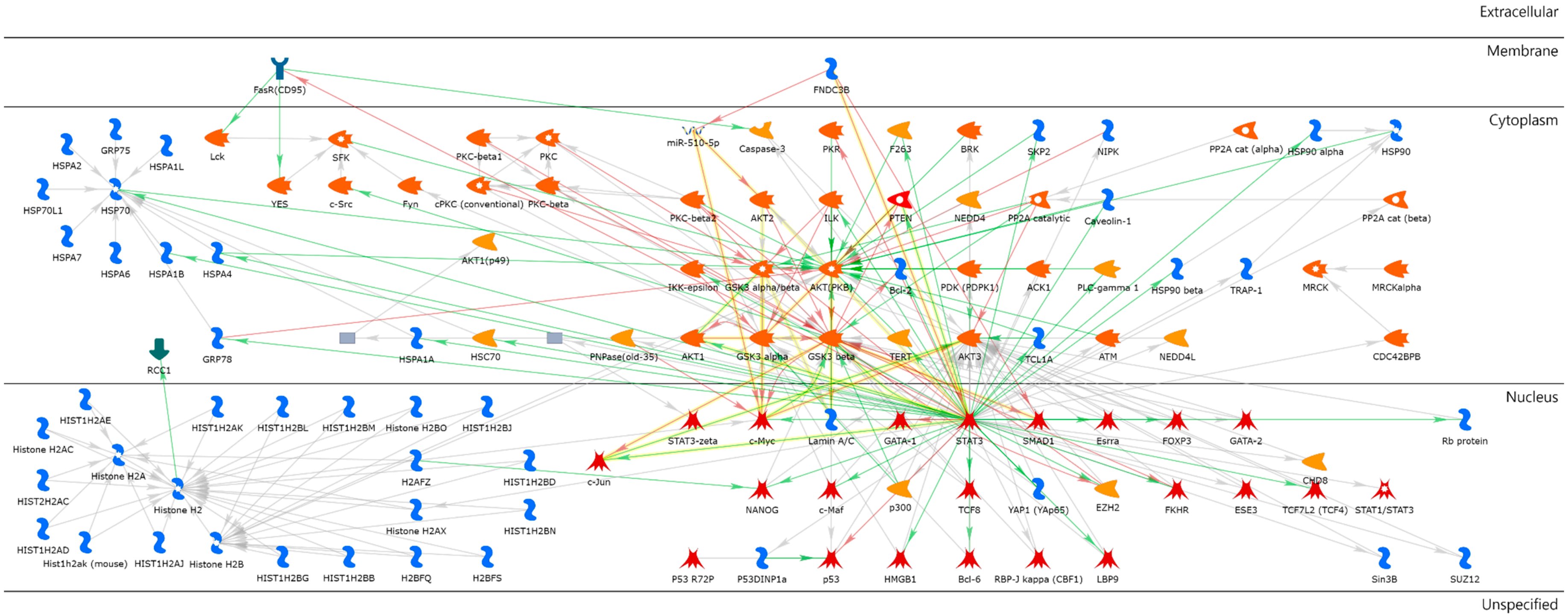

Figure S2. Network Analysis Results of FNDC3B Related to STAT3 and Akt. STAT3 and Akt were selected based on their relevance to FNDC3B. STAT3 was found to be most closely related to FNDC3B in the sole FNDC3B network analysis (Figure 5a), while Akt was identified as the most affected by FNDC3B in the proteome profiler phospho-kinase array and western blot (Figures 5b and 5c). Using the Metacore database, an integrated pathway bioinformatics analysis platform, a network analysis was conducted. The network was examined using an auto-expand building algorithm comprising 50 nodes and employing canonical pathways. The arrows indicate the following: green for positive/activation effects, red for negative/inhibition effects, and gray for unspecified. The networks of interest are highlighted in yellow. FNDC3B: fibronectin type III domain containing 3B; STAT3: signal transducer and activator of transcription 3.

Table S1: Primer sequences used for real-time quantitative reverse transcription polymerase chain reaction.

Gene	Forward (5' – 3')	Reverse (5' - 3')
**FNDC3B**	GGTGAGGGCTCTGAATGATG	CTCCCAACCCACTAAGACAC
**Survivin**	GGCCCAGTGTTTCTTCTGCTT	GCAACCGGACGAATGCTTT
**Nestin**	AACAGCGACGGAGGTCTCTA	TTCTCTTGTCCCGCAGACTT
**CD44**	GCTATTGAAAGCCTTGCAGAG	CGCAGATCGATTTGAATATAACC
**α-SMA**	CCGACCGAATGCAGAAGGA	ACAGAGTATTTGCGCTCCGAA
**GAPDH**	TGGGTGTGAACCATGAGAAG	AGTCCTTCCACGATACCAAAG

α-SMA: alpha-smooth muscle actin; FNDC3B: fibronectin type III domain containing 3B; GAPDH: glyceraldehyde 3-phosphate dehydrogenase.

Table S2: Z-values related to FNDC3B expression in the database of glioblastoma patients.

Dataset	Number of samples	Z-value	Dataset	Number of samples	Z-value
**PMID-18772890-TCGA**	203	-2.8114	**GSE43378**	32	-1.5307
**GSE83300**	50	-3.2472	**GSE4271.HG-U133B**	56	0.1654
**GSE83294.HG-U133B**	59	1.5402	**GSE4271.HG-U133A**	56	-0.5315
**GSE83294.HG-U133A**	59	0.4735	**GSE42669**	55	0.3160
**GSE79671**	36	-1.6375	**GSE33331**	21	1.7564
**GSE7696**	80	0.9558	**GSE30472**	29	-0.9660
**GSE74187**	60	-1.2472	**GSE26576**	25	-1.3669
**GSE72951**	110	-0.9745	**GSE1993**	39	1.6294
**GSE61335.HG-U133B**	44	-2.1742	**GSE18166.GPL9190**	81	0.3089
**GSE61335.HG-U133A**	44	-2.2186	**GSE13041.HG-U133_PLUS_2**	27	-0.3445
**GSE4412.HG-U133B**	59	1.5402	**GSE13041.HG-U133A**	90	-0.9041
**GSE4412.HG-U133A**	59	0.4735			

FNDC3B: fibronectin type III domain containing 3B.

Table S3: Sequences of small interfering RNAs created to investigate the functions of FNDC3B.

siRNA	No.	Sense(5' → 3')	Antisense(5' → 3')
Control	-	AAUUCUCCGAACGUGUCACGUUU	AAACGUGACACGUUCGGAGAAUU
FNDC3B	1	UUCCAGAGGGAUUUGGUCG	CGACCAAAUCCCUCUGGAA
	2	UACACUGUGUCUGUUGCAC	GUGCAACAGACACAGUGUA
	3	AUGAGUUUGACAUACAGAG	CUCUGUAUGUCAAACUCAU
